# RESILIENCE AMONG RESIDENTS, THEIR FAMILY, AND STAFF OF A CONTINUING CARE RETIREMENT COMMUNITY

**DOI:** 10.1093/geroni/igac059.2973

**Published:** 2022-12-20

**Authors:** Justine Sefcik, Yeji Hwang, Zachary Hathaway, Martha Coates, Shelby Hufnal, Isabella Stoll, Rose Ann DiMaria-Ghalili, Kathleen Fisher

**Affiliations:** Drexel University, Philadelphia, Pennsylvania, United States; Drexel University, Philadelphia, Pennsylvania, United States; Drexel University, Philadelphia, Pennsylvania, United States; Drexel University, Philadelphia, Pennsylvania, United States; Drexel University, Philadelphia, Pennsylvania, United States; Drexel University, Philadelphia, Pennsylvania, United States; Drexel University, Philadelphia, Pennsylvania, United States; Drexel University, Philadelphia, Pennsylvania, United States

## Abstract

There is emerging literature about older adults’ experience of loneliness during the COVID-19 pandemic in long term care (LTC) facilities due to isolation protocols. Additionally, staff challenges while providing care for older adults in LTC has also been highlighted. While the literature emphasizes negative pandemic experiences, a gap exists with understanding resilience during the pandemic in LTC settings. The aim of this qualitative descriptive study was to explore the experience of resilience in a Continuing Care Retirement Community (CCRC) among residents, their family members, and staff. We conducted 19 in-person interviews and 1 via Zoom in fall 2021 with 5 residents (65 and older), 5 family members, and 10 staff (e.g., administrators, nurses, nursing assistants). A conventional content analysis was employed. While we did hear how the pandemic had a negative impact on everyone, the main themes of resilience identified were: 1) overcoming the pandemic together (sense of community); 2) experience and adaptation (over time being able to adapt to the disruption in their life); 3) staying safe (engaging in precautions and self-care strategies); and 4) positivity (mindset of getting through anything and relying on spirituality). Study findings can inform CCRC administrators on how to support residents, their family, and staff during future pandemics and other challenging times that may disrupt normal routines.

